# Retrieval Algorithms for Road Surface Modelling Using Laser-Based Mobile Mapping

**DOI:** 10.3390/s8095238

**Published:** 2008-09-01

**Authors:** Anttoni Jaakkola, Juha Hyyppä, Hannu Hyyppä, Antero Kukko

**Affiliations:** 1 Finnish Geodetic Institute, Department of Remote Sensing and Photogrammetry, P.O. Box 15, 02431 Masala, Finland; E-Mails: juha.hyyppa@fgi.fi; antero.kukko@fgi.fi; 2 Helsinki University of Technology, Institute of Photogrammetry and Remote Sensing, P.O. Box 1200, 02015 Espoo, Finland; E-mail: hannu.hyyppa@tkk.fi

**Keywords:** Mobile mapping, road surface, kerbstone, modelling, laser scanning

## Abstract

Automated processing of the data provided by a laser-based mobile mapping system will be a necessity due to the huge amount of data produced. In the future, vehicle-based laser scanning, here called mobile mapping, should see considerable use for road environment modelling. Since the geometry of the scanning and point density is different from airborne laser scanning, new algorithms are needed for information extraction. In this paper, we propose automatic methods for classifying the road marking and kerbstone points and modelling the road surface as a triangulated irregular network. On the basis of experimental tests, the mean classification accuracies obtained using automatic method for lines, zebra crossings and kerbstones were 80.6%, 92.3% and 79.7%, respectively.

## 1. Introduction

### 1.1. Background

Accurate and intelligent up-to-date static roadside information is needed for road and street planning and engineering, starting with the planning phase and ending with the rehabilitation or maintenance phase. The planning phase requires a reasonably accurate digital elevation model, which preferably should include 3D object information. The DEM and 3D models are used for the planning of mass analysis, visual aspects of landscape design and environmental impact assessment. This kind of information can be obtained with airborne laser scanning at low altitudes.

When the road has been constructed, documentation of road information is needed for an increasing number of other applications, such as noise modelling, road safety, road maintenance, location-based services, and car and pedestrian navigation. Documentation of the road environment includes documentation of both road geometry and road environment.

Road geometry means the parameters used for the geometric design of roads, such as design speed, stopping sight distance, passing sight distance, line of sight, number of lanes, lane width, side foot path for pedestrians, longitudinal and transverse slope, road pavement materials, etc. Recently built roads follow modern geometric design parameters, and most of this information is already available, but for the oldest roads these data, which are required mostly for upgrading or assessing the quality of the roads, are unavailable or unusable. By road environment we refer to the vicinity of the road on both sides of the road, including buildings, trees, vegetation, electricity, traffic signs, traffic light poles and other objects [1]. We expect that the geometry of this kind of road environment can best be obtained either using airborne or vehicle-based laser scanning or using an integrated approach combining both systems. Supported with imaging sensors, the documentation can be completed with texture information, traffic sign semantics, etc.

### 1.2. Overview

The need for three-dimensional data from road environments is rapidly growing as more and more location-based services are emerging. Consumer-grade navigation systems are beginning to move towards presenting data with 3D models rather than conventional 2D maps. Vehicle-based laser scanning, hereinafter mobile mapping, is an efficient way of collecting spatial information concerning roads and their surroundings. Recent papers on laser-based mobile mapping systems (MMS) have been published by El-Sheimy [2], Talaya *et al.* [3], Tao [4], Zhao and Shibasaki [5]. Concerning industrially manufactured MMS, both Optech (Lynx system, 1 to 4 laser scanners, each having a 100 kHz PRF) and 3D Laser Mapping (Streetmapper, 4 scanners, 10 kHz each) provide commercial systems for emerging markets. However, the amount of data produced by such systems is huge, and manual processing of the data is very time-consuming, which prompts a need for automated methods that decrease the amount of manual work required to produce accurate 3D models. At present, it is possible to use software and methods developed for terrestrial and airborne laser scanning, but due to different scanning geometry, different point density and the fast processing needed, algorithms for MMS data processing need to be developed separately.

Only a few fully automated methods for classifying and modelling the road environment on the basis of laser data provided by a mobile mapping system have been described. Früh, Jain and Zakhor [6] proposed an automated method for modelling building façades. Goulette *et al.* [7] presented a method for automatically extracting the road surface, trees and vertical surfaces from the laser scans. The methods and models used are quite simple, and the modelled objects lack detail. Yu *et al.* [8] studied the use of laser scanners and video cameras in creating detailed models of the road surface, and their models seem geometrically quite accurate, down to small cracks in the road surface. Zhao and Shibasaki [5] used line cameras and laser scanners to produce textured planar surfaces.

Many methods using stationary terrestrial scanning have been proposed (e.g. [9-12]). In some applications, the methods can be directly applied to mobile mapping, but in some cases, the data produced by mobile mapping are too different from the data provided by stationary scanning. Two main differences between stationary and mobile scanning are the evenness of the data and the perspective. In mobile mapping, the point cloud is evenly distributed along the driving direction, and the viewing direction to the target remains approximately constant.

In this paper, we propose an automatic retrieval method to model a road, consisting of kerbstones and road surface along with the paintings, including zebra crossings and roadside parking spaces, based on a dense point cloud produced by vehicle-based laser scanning. The curbstones and markings on the road are segmented by applying image-processing algorithms to the intensity and height images, and the pavement is modelled as a triangulated irregular network based on the point cloud. A comparison with previous work shows that Goulette *et al.* [7] modelled the road surface as a flat plane as opposed to our TIN, which better describes the irregularities in the surface. Yu *et al.* [8] used a densified point cloud from video cameras, whereas we only use a single laser scanner with a profile spacing of c. 20-50 cm. The previously mentioned methods focused on finding the geometry either as simple planes or as a highly detailed model, whereas our study concentrates more on classifying and modelling the road surface.

### 1.3. FGI Roamer

The MMS data were collected with the FGI Roamer mobile mapping system developed at the Finnish Geodetic Institute. The Roamer consists of a Faro LS 880 laser scanner with a measurement frequency of 120 kHz and a NovAtel HG1700 SPAN_58_ INS system. With slightly modified hardware for the standard FARO LS, it provides so-called tunnel mode, or profile measurements, synchronized with external positioning and data logging systems. This information is needed to derive the position and attitude information for each 3D point produced by the laser scanner. The mirror rotation frequency, or scan rate of the scanner on the Roamer can be set to 3-30 Hz, thus giving a vertical angular resolution of 0.0096-0.096 degrees (0.17-1.7 mrad), respectively. Corresponding point spacing at a typical scanning range of 15 metres in road mapping is thus 2.5-25 mm in the scanning plane. [13]

### 1.4. Modelling process

**Figure 1.**
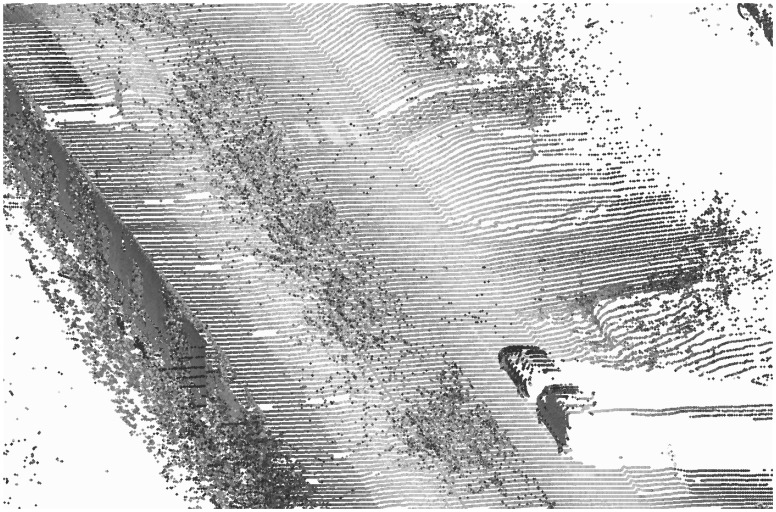
Part of the original point cloud showing a zebra crossing and a parking space line.

The modelling process consisted of several steps. First, we classified the points belonging to the painted markings on the road surface based on the intensity level, and then we continued to find the kerbstones from the height image. Finally the pavement was modelled as a TIN. Part of the point cloud used as a starting point for the modelling process is shown in Figure 1.

The data used to model road markings and kerbstones were formatted as raster images. This allowed us to use raster image-processing algorithms that are very efficient compared with point cloud processing. The raster images created included intensity image and height image. The pixel size of the images varied as a function of the measurement distance and driving speed but was usually between 10 cm^2^ and 100 cm^2^ with measurement distances of 3 m to 13 m and driving speeds of 20 km/h to 50 km/h. The typical pixel size on the road of the dataset shown in this article was about 56 cm × 0.4 cm.

The intensity image used to model the road markings, as seen in Figure 4, was constructed directly from the measurements of the laser scanner. Each column in the intensity image corresponds to a profile measured by a single rotation of the mirror. Similarly, the rows in the image correspond to consecutive measurements along the profile. Curbstones were modelled using a height image constructed from the z coordinate values.

Because there was no use for the upper parts of the point cloud mostly consisting of buildings and sky, we cropped the images automatically. The boundaries for point cloud cropping were selected by finding points in the median intensity curve that corresponded to the 90% level of the original intensity value of the point 0, which is the measurement point right behind the measurement platform. In our example in Figure 3, the point 0 had an intensity value of about 1,550, which caused the boundary value to be set at about 1,400.

## 2. Modelling of road markings

### 2.1. Modelling process

**Figure 2.**
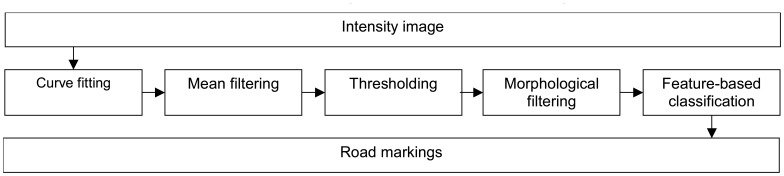
Modelling process of the road markings.

The process of modelling the markings consisted of several steps. We first pre-processed the data to even out the changes in intensity values and then thresholded the image. After thresholding, we applied morphological operations to make sure that segments were of an appropriate size. Then we classified the segments as zebra crossings and other lines according to their properties. The process used to extract the road markings is shown in Figure 2.

### 2.2. Pre-processing and segmentation

**Figure 3.**
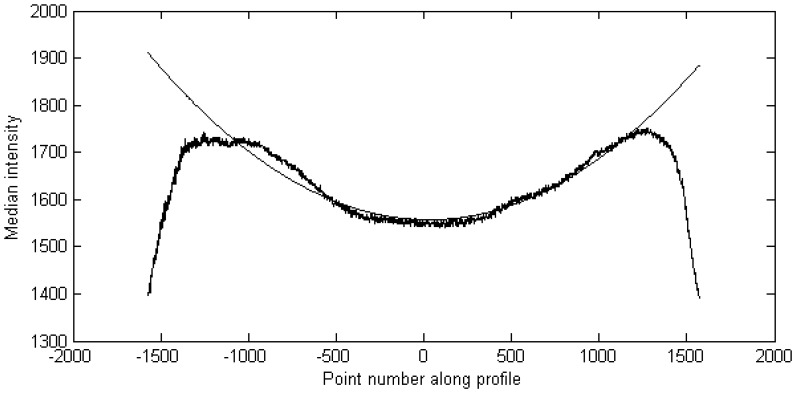
Fitted intensity curve.

The segmentation of road markings began with pre-processing of the image. First, we reduced the variance of the measured intensity value along the profile by fitting a second order curve to the median intensity measurements along the profile. This reduced the fading of the intensity towards the edges of the road caused by the longer distance from the laser scanner and the larger angle of incidence. In the future, however, better radiometric calibration methods for the intensity should be developed. Only values lying between the peaks on both sides of the centre of the profile were used in fitting. The fitted curve is shown in Figure 3.

**Figure 4.**
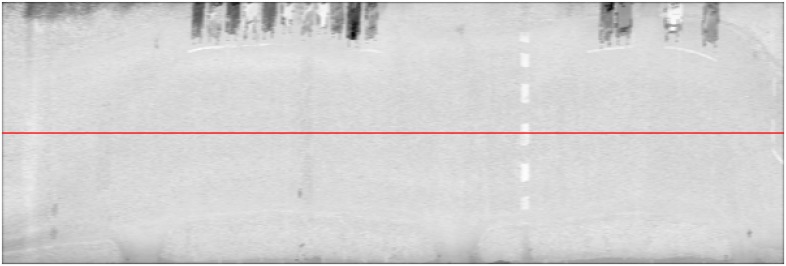
Pre-processed intensity image showing the centreline corresponding to pixel 0 of each profile in red.

We then smoothened the radiometrically corrected intensity image by applying a 3-by-5 average filter to the image. This helped remove noise and select correct values for individual erroneous measurements. We found out that the noise was best filtered using a 3-by-5 average filter without losing too much detail. The resulting pre-processed intensity image is shown in Figure 4.

The curve fitting explained earlier scaled the intensity values so that the mean value was close to 1. Therefore we could use constant threshold values. To extract the road markings, we applied a threshold of 1.05 to the intensity image. The threshold value could be selected to be constant because the intensity values had been normalized in the fitting of the intensity curve. The curve fitting caused the pavement to get an intensity value of approximately 1.0 and thus the threshold value of 1.05 required that the road paintings had to be at least 5 % brighter than the average pavement.

After thresholding, we applied a series of morphological operations. We first opened the image with a 3-by-15 mask, then closed it with a 5-by-9 mask and finally opened the image with a 5-by-1 mask, i.e.

(1) G=((F∘A)•B)∘C

where G is the resulting image, F is the original thresholded image and A, B and C are masks of sizes 3-by-15, 5-by-9 and 5-by-1. The first opening removed small regions that were caused by areas with intensity value near the threshold value. The closing of the image was used to connect the line segments that belonged to the same line. The final opening removed the lines that were perpendicular to the driving direction; this was done because they could impair the classification result. At this point, we did not even try to model the lines perpendicular to the scanner trajectory, because with a profile spacing of about 50 cm the scanner is only able to see one in every five such lines if we assume a line width of about 10 cm.

### 2.3. Classification of road markings

Painting segments found in the segmentation phase were classified using properties calculated for each 4-connected segment of the image. The properties determined for each segment were: area, area of the bounding box, and orientation. The orientation was defined as the direction of maximum variance, which could be calculated using the Karhunen-Loève transformation.

For a segment to be classified as a line, the area of the segment had to be between 200 and 10,000 pixels. With a driving speed of about 30 km/h and a mirror rotation rate of 15 Hz, the profile spacing was about 56 cm, and the point spacing along the profile at a typical measurement range of 5 m was 4 mm. With these assumptions, we arrived at a pixel size of 22 cm^2^/pixel, meaning that the range of 200 to 10,000 pixels corresponded to a real-world area of 0.45 m^2^ to 22 m^2^. These requirements caused small erroneous segments and large segments usually consisting of the grass areas along the road to be left out.

A line was classified to be part of a zebra crossing if the area of the segment was at least 70% of the area of the bounding box and the orientation was above 88°. The bounding box rule required the segment to be rectangular, and the orientation requirement ensured that the segment was wider than it was long in terms of pixels. To make sure that isolated patches of white paint were not included as zebra crossings, we required that at least four segments classified as possible zebra crossings had to be alongside each other. As the edge of the marking can be blurry, we finally cleaned it up by thresholding the zebra crossing points with an intensity threshold of 1.08.

A line was classified as an ‘other line’, e.g. parking space line, if it had an orientation of less than 80°. Furthermore, we required that the variance in the direction of minimum variance be below 0.003. Again, to clean up the edges of the lines, we removed all the points with an intensity level below 1.06.

## 3. Processing of Curbstones

**Figure 5.**
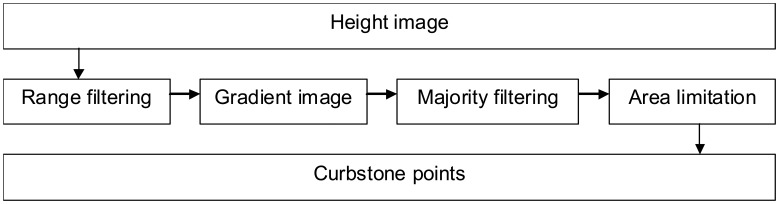
Curbstone modelling process.

The process of modelling curbstones was relatively straightforward, as it was based on the height gradient along the scanned profile. The gradient information was then processed into segments that could be considered kerbstones. Figure 5 shows the modelling process of the curbstones.

The modelling of curbstones began by extracting them from the height image. We first calculated the height difference in the direction of the scan line and required the absolute value of the gradient to be between 0.002 m and 0.2 m per pixel. This gave us pixels that were neither horizontal nor vertical, eliminating flat road surfaces, cars and trees. To be accepted as a possible curbstone point, a pixel had to have at least 60 pixels in the 5-by-25-pixel neighbourhood with gradients in the range specified above. This step was necessary because the height measurements were noisy and thresholding the height gradient produced areas with several small holes. Because the majority filtering slightly diminished the area, we dilated the region with a 3-by-11 rectangular structuring element. Finally, we required that these candidate pixels form a 4-connected region with an area of at least 100 pixels. If we assume a curbstone width of 15 cm, this translates into a length of 1.5 m.

## 4. Road surface triangulation

**Figure 6.**
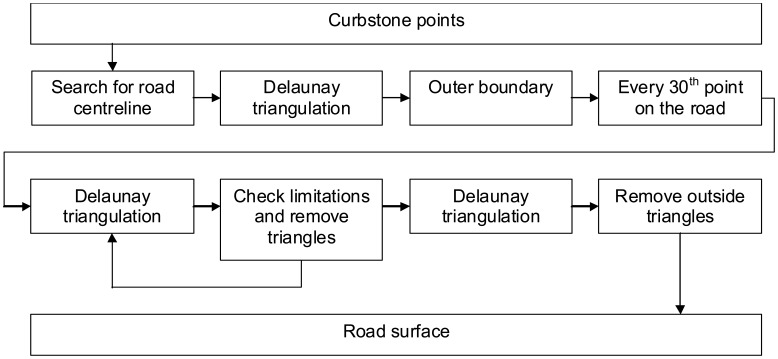
Road surface modelling process.

A road surface model was created by first finding all the points in the point cloud that lay on the road area. Then we created a TIN and iteratively shaped the TIN into a smooth surface by setting slope and edge length constraints. The modelling process of the road surface is shown in Figure 6.

In preparation for triangulating the road surface, we found the outer bounds of the pavement based on the previously modelled curbstones. A Delaunay triangulation was formed from the curbstone points to remove points lying outside the road surface. We found the approximate trajectory of the laser scanner by taking the x and y coordinates of the point right behind the measurement platform, i.e. point 0 of each scan line, and removed all the triangles that did not cross this trajectory. Whether a triangle crossed a trajectory segment could be easily checked using equations

(2) $$a=\frac{\left(x_{4}-x_{3})(y_{1}-y_{3})-(y_{4}-y_{3})(x_{1}-x_{3}\right)}{\left(y_{4}-y_{3})(x_{2}-x_{1})-(x_{4}-x_{3})(y_{2}-y_{1}\right)}$$

(3) $$b=\frac{\left(x_{2}-x_{1})(y_{1}-y_{3})-(y_{2}-y_{1})(x_{1}-x_{3}\right)}{\left(y_{4}-y_{3})(x_{2}-x_{1})-(x_{4}-x_{3})(y_{2}-y_{1}\right)}$$

where *x_1_*, *y_1_*, *x_2_* and *y_2_* are the coordinates of the trajectory segment endpoints and *x_3_*, *y_3_*, *x_4_* and *y_4_* are the coordinates of the triangle edge [14]. If both *a* and *b* were between 0 and 1, the lines crossed. The outer bounds of the pavement were found by removing all the inner edges of the triangulation by traversing the outer bound vertex by vertex. As an initial set of pavement points, we selected every 30th point from the original point cloud that lay within the boundaries.

**Figure 7.**
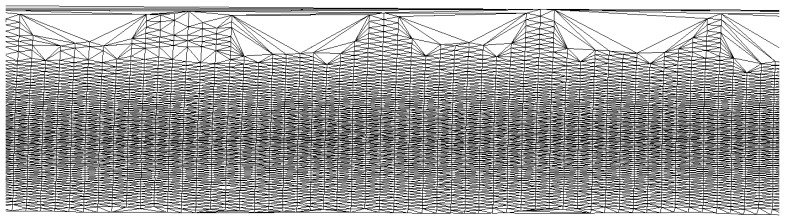
Part of the final road surface triangulation.

From the initial pavement points, we created a Delaunay triangulation to represent the surface. For each triangle, we calculated the height difference between the highest and the lowest point and divided it by the length of the longest edge, which we used to estimate the slope of the triangle. If a triangle had a slope value greater than 20% along the longest edge, the highest point of the triangle was removed. After removing all the triangles failing to meet the requirement, we formed a new triangulation and repeated the point removal step. This was done repeatedly for as long as there were points that did not satisfy the requirements. Part of the final triangulation of the road surface is shown in Figure 7. The gaps that can be seen in the triangulation in figure 7 were caused by parked cars.

## 5. Results

**Figure 8.**
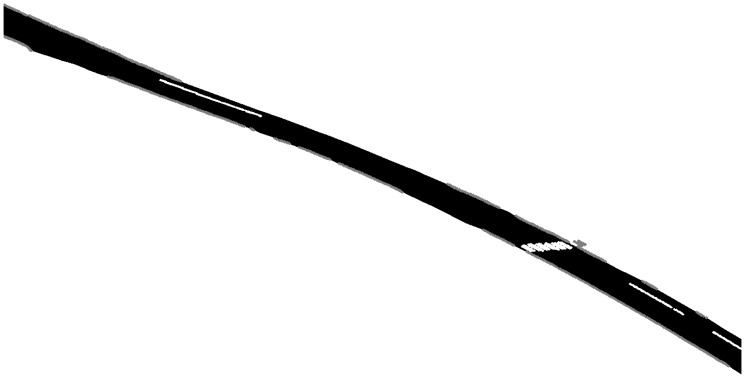
Final road model with grey curbstones and white road markings corresponding to the parking space lines and zebra crossings.

Based on visual inspections and field checking, the proposed methods were able to find most of the kerbstones that were visible to the laser scanner along with the road markings that were clearly distinguishable. Parts of the parking space lines were so worn that even a human operator could not have classified them outright. The zebra crossing was detected as expected. The final model of the road with kerbstones, zebra crossings, parking space lines and the triangulated road surface is shown in Figure 8. It can be seen that at the end of the zebra crossing there is a group of curbstone points. These curbstones are correctly classified, even though they actually belong to a stairway.

The completeness and correctness of the produced model were assessed with a ground reference obtained using manual classification of the dataset. The acquired modelling accuracies using the same data are shown in Table 1. Based on the intensity image it is often unclear where the real edge should be, and therefore part of the error could be caused by the ambiguousness of the line edges in the reference data. Some short line segments were completely missed because they were too short to be found by the algorithm, as it assumes a minimum area of 200 pixels, which corresponds to a line length of about 4.5 m.

## 6. Conclusions and future work

In this paper, fully automatic methods for processing mobile mapping data for road surfaces are proposed. This is vital since such vehicle-based laser scanning allows fast processing of long corridors, e.g. thousands of kilometres of road. Such information will be needed in future 3D road environment mapping, e.g. in automatic updating of the 2D and 3D navigation data for consumer and business applications. Visual experiments confirmed that the methods developed identified most of the features. The zebra crossing and the curbstones were identified as expected, and the parking space lines were classified correctly where possible. More detailed analysis showed mean accuracies of about 80% or better for lines, zebra crossings and curbstones.

Future studies will demonstrate the completeness and correctness of such methods with larger experimental sample. At present, large MMS data based on dense laser scanning data do not yet exist. The methods will be further developed to be applicable for road crossings, dashed centrelines and other kinds of road environment. Also median islands and pedestrian refugees cause problems to the current methods.

## Figures and Tables

**Table 1.** Assessment of modelling accuracy.

**Target**	**Completeness**	**Correctness**	**Mean accuracy**
Lines	86.6%	74.6%	80.6%
Zebra crossings	95.1%	89.5%	92.3%
Curbstones	73.9%	85.6%	79.7%
